# Climate change mitigation opportunities based on carbon footprint estimates of dietary patterns in Peru

**DOI:** 10.1371/journal.pone.0188182

**Published:** 2017-11-16

**Authors:** Ian Vázquez-Rowe, Gustavo Larrea-Gallegos, Pedro Villanueva-Rey, Alessandro Gilardino

**Affiliations:** 1 Peruvian LCA Network, Department of Engineering, Pontificia Universidad Católica del Perú, San Miguel, Lima, Peru; 2 Department of Chemical Engineering, Institute of Technology, Universidade de Santiago de Compostela, Santiago de Compostela, Galicia, Spain; TNO, NETHERLANDS

## Abstract

Food consumption accounts for an important proportion of the world GHG emissions per capita. Previous studies have delved into the nature of dietary patterns, showing that GHG reductions can be achieved in diets if certain foods are consumed rather than other, more GHG intensive products. For instance, vegetarian and low-meat diets have proved to be less carbon intensive than diets that are based on ruminant meat. These environmental patterns, increasingly analyzed in developed nations, are yet to be assessed in countries liked Peru where food purchase represents a relatively high percentage of the average household expenditure, ranging from 38% to 51% of the same. Therefore, food consumption can be identified as a potential way to reduce GHG emissions in Peru. However, the Peruvian government lacks a specific strategy to mitigate emissions in this sector, despite the recent ratification of the Paris Accord. In view of this, the main objective of this study is to analyze the environmental impacts of a set of 47 Peruvian food diet profiles, including geographical and socioeconomic scenarios. In order to do this, Life Cycle Assessment was used as the methodological framework to obtain the overall impacts of the components in the dietary patterns observed and primary data linked to the composition of diets were collected from the Peruvian National Institute for Statistics (INEI). Life cycle inventories for the different products that are part of the Peruvian diet were obtained from a set of previous scientific articles and reports regarding food production. Results were computed using the IPCC 2013 assessment method to estimate GHG emissions. Despite variations in GHG emissions from a geographical perspective, no significant differences were observed between cities located in the three Peruvian natural regions (i.e., coast, Andes and Amazon basin). In contrast, there appears to be a strong, positive correlation between GHG emissions and social expenditure or academic status. When compared to GHG emissions computed in the literature for developed nations, where the average caloric intake is substantially higher, diet-related emissions in Peru were in the low range. Our results could be used as a baseline for policy support to align nutritional and health policies in Peru with the need to reduce the environmental impacts linked to food production.

## Introduction

Peru is one of the main international destinations for culinary holidays [1–3]. The variety of endemic edible products from the highlands, as well as from the Peruvian Amazon, together with a thriving gastronomic industry in the city of Lima has made Peru a leading nation in terms of innovation in the food sector. Despite the buoyancy of its food sector, as well as high economic growth rates since the year 2000, there are still severe cases of child malnourishment in many regions, and in impoverished districts average diets are based on a very limited variety of food basket products [4–5]. According to the World Food Programme, over 4% of Peruvians still lived, as of 2014, in extreme poverty, and approximately 5.2 million people are highly exposed to food insecurity [6], in a country located in a high seismic activity zone, and with cyclical droughts and floods [7–8].

To make matters worse, the average Peruvian diet, lacking in the consumption of fruit and vegetables, is animal-based, mainly poultry, and with a high consumption of staples and cereals [9]. Such animal protein-based diets are known, from an environmental perspective, for their higher energy consumption and greenhouse gas (GHG) emissions [10–13]. Therefore, several studies analyzed the possibility of social shifts towards more environmentally sustainable diets in many nations with a lower reliance on animal (mainly beef and other ruminant) protein [12]. For instance, Scarborough and colleagues [2014] identified a positive relationship between GHG emissions and the intake of animal-based products in UK diets [13]. Moreover, a recent study in Germany suggests that 11% of the population is now full-time vegan or vegetarian with the percentage rising to 52% if people that avoid eating meat at least three times per week are included [14].

As regards Peru there is no official data reporting the percentage of vegans or vegetarians in the population. Although it is noticeable that there is a growing population of vegetarian restaurants in the city of Lima and other touristic destinations [15], changes towards non-animal based diets tend to occur at faster paces in developed nations [16]. Therefore, it is not feasible to expect a rapid shift of vast sectors of the population to environmentally-sustainable diet options.

It is reasonable to acknowledge that most research on sustainable diets has been performed in developed nations [13, 17–18], despite the fact that food growth rates are occurring at higher rates in rapidly developing countries, such as Peru, together with obesity and chronic cardiovascular diseases [19–21]. In this context, it should be noted that although mitigating environmental impacts, namely GHG emissions, was considered a worldwide priority according to the Millennium Development Goals [22], eradication of malnourishment and hunger is considered a more important priority in the short- and mid-term according to the new 17 Sustainable Development Goals [23]. A certain degree of relationship between the two goals has been identified to the extent that climate change may constitute a potential obstacle towards a world with zero hunger [24]. In fact, Lobell et al. [25] note that basic food products in the Peruvian diet, such as wheat and rice, and to a lesser extent, potatoes, may experience significant production reductions in the Andean region by 2030 due to climate change.

In this context, Peru is one of the countries that have already ratified the Paris Accord, with a relatively ambitious objective of reducing environmental impacts by at least 31% in 2030 as compared to a business-as-usual scenario [26]. Although in the case of Peru the GHG emissions linked directly to the food sector are yet to be computed in detail, studies elsewhere show that the food sector can represent up to 15–30% of national GHG emissions [27–28]. Moreover, this proportion can reach up to 21–47% if land use changes are considered [28]. GHG emissions linked to individual food products have been analyzed sparingly in Peru through a set of Life Cycle Assessment (LCA) studies. However, most of these have focused on the GHG emissions linked to the production of fishmeal and fish oil [29], agro-exports to other continents [30–31], or alcoholic beverages [32]. In fact, only a few studies have focused on the production of nationally-consumed food products, including dairy products [33] and fruits [34].

Consequently, there is an imperative need to improve the available information linked to the environmental impacts associated with the consumption of food in Peru. To date, food intake has been addressed from a social and health perspective and used only to build economic and social indicators, but it needs to incorporate the environmental pillar in order to be able to understand how increased food consumption, mainly due to the nation’s economic growth, will impact different environmental problems [35]. In other words, Peru has to align its food policy with broader environmental issues, in order to tackle malnourishment, extreme poverty or diet-related diseases, while avoiding the replication of unhealthy and unsustainable diets that have developed in the United States or Europe [20].

In this context, the main objective of this study is to calculate the emission of GHGs linked to different geographical and socioeconomic diet profiles in Peru, with the aim of mapping the environmental impacts that these generate. Moreover, a further objective is to link socioeconomic trends in Peruvian society with these values, analyzing what public policies may be implemented to guarantee the environmental sustainability of the average diet. The results of this study are expected to be of utility in policy-making in Peru as a way to integrate sustainable approaches with food security.

## Materials and methods

The *Pontificia Universidad Católica del Perú*'s Ethical Committee approved the WALAYA project prior to the beginning of activities.

### Data acquisition

Data on food consumption in Peruvian households were obtained from a national survey performed by the Peruvian Statistics Unit (i.e., INEI), ENAPREF [36], which aggregates the most recent purchase of food patterns in households between May 2008 and April 2009 for a total of 36,234 dwellings. Of these, 34,698 were sampled in urban environments, which led to the decision to focus mainly on cities [37].

Based on these data, a set of 47 diet scenarios were constructed, as depicted in Table 1, taking into consideration average diets in Peruvian cities, socioeconomic quintiles and education. It should be noted that Callao (the second largest city in Peru based on the number of inhabitants) is not included in Table 1 since it is part of Metropolitan Lima (henceforth referred to as *Lima Metropolitana*). Therefore, all reference to *Lima Metropolitana* will actually refer to the urban agglomeration of the two cities. All other regional capitals were also included in the study, except for the regions of Loreto and San Martín. For the former, the city of Iquitos (3°44’S; 73°15’W) was excluded from the study due to its particular characteristics in an impassable area of the Amazon basin, constituting as it does the largest continental city on Earth that is not accessible by road from other major cities. Therefore, most of the food products that are airfreighted into the city from Lima or freighted by river from Brazil are subject to high “food miles” GHG emissions as compared to other cities [38]. For the latter, the most populous city of the San Martin region, Tarapoto (6°29’S; 76°22’W), was included rather than Moyobamba (6°02’S; 76°58’W), the region’s capital. In addition, the city of Chimbote (9°04’S; 78°35’W), the most populous in the region of Áncash, was included in the study.

**Table 1:** Diet profiles modelled for different geographical and socioeconomic scenarios in Peru.

Scenario types	Description
***Geographical***	
• National	Average Peruvian diet
• Urban	Metropolitan Lima^a^
	City of Abancay (Apurímac)^b^
	City of Arequipa (Arequipa)
	City of Ayacucho (Ayacucho)
	City of Cajamarca (Cajamarca)
	City of Chachapoyas (Amazonas)
	City of Chiclayo (Lambayeque)
	City of Chimbote (Áncash)
	City of Cusco (Cusco)
	City of Huancavelica (Huancavelica)
	City of Huancayo (Junín)
	City of Huánuco (Huánuco)
	City of Huaraz (Áncash)
	City of Ica (Ica)
	City of Moquegua (Moquegua)
	City of Cerro de Pasco (Pasco)
	City of Piura (Piura)
	City of Pucallpa (Ucayali)
	City of Puerto Maldonado (Madre de Dios)
	City of Puno (Puno)
	City of Tacna (Tacna)
	City of Tarapoto (San Martín)
	City of Trujillo (La Libertad)
	City of Tumbes (Tumbes)
***Socioeconomic***	
	Quintile I^c^
	Quintile II^c^
	Quintile III^c^
	Quintile IV^c^
	Quintile V^c^
***Educational***	
	Head of household with an academic degree
	Head of household with high school degree or lower

^a^ Metropolitan Lima includes the city of Callao.

^b^ Names in brackets represent the region to which each city belongs.

^c^ Quintiles are based on the economic expenditure of the households, with Quintile I representing the segment with lowest expenditure and Quintile V representing the segment with highest expenditure. Quintiles have been computed independently for the following geographical divisions: a) Metropolitan Lima; b) Urban coastal Peru; c) Urban Andean Peru; d) Urban Amazonian Peru.

In addition to the data per geographical location described above, a series of socioeconomic and educational dietary patterns were also extracted from ENAPREF. On the one hand, data on diets based on household expenditure cut-off points for the population were modelled. For this, quintiles based on household expenditure were used, in which the first quintile represents the lowest fifth of the population in terms of consumption (1–20%). These quintiles were available, as shown in Table 1, for *Lima Metropolitana* and for the mean urban areas in the Peruvian Coast, in the Andes and in the Amazon basin. On the other hand, a division was made between households based on the academic level of the head of the family (i.e., with or without a University degree).

It should be noted that despite the fact that the survey was conducted at a household level, the results of food purchase were reported per capita considering the number of members in each dwelling [36]. Another point that should be highlighted is the fact that food purchase only represents what is acquired for preparation and/or consumption in the household, whereas food consumed in restaurants, canteens or in the streets, referred to as food-away-from-home (FAFH), is excluded from this list.

### Food and caloric intake

Besides the purchase of food products in Peruvian households, the Peruvian Tables of Food Composition were used to calculate the caloric intake linked to these purchases [39]. In addition, the individual food products were divided into 15 categories, following the structure provided in ENAPREF [36], which roughly represent traditional food groups (e.g., cereals, dairy products, vegetables, etc.), as shown in Table 2.

**Table 2:** Food categories included in the study based on the division provided by the *Encuesta Nacional de Prespuestos Familiares* (ENAPREF, 2012).

	Food category	Types of food products included
1	Cereals	Rice, wheat, barley, oats…
2	Tubers	Potato, sweet potato, native Peruvian tubers…
3	Vegetables	Tomatoes, lettuce, carrots, celery…
4	Fruits	Bananas, apples, strawberries, mangoes, oranges, papaya, grapes…
5	Oils	Unspecified
6	Dairy	Milk, yogurt, cheese…
7	Fish and shellfish	Divided in fresh marine, canned marine, river fish and dried or salted.
8	Chicken and other poultry	Chicken, eggs and others.
9	Legumes	Peas, beans, lentils…
10	Red meat	Beef, offal, lamb and pork
11	Ice cream	Unspecified
12	Soft drinks	Soda and juices
13	Food items rich in sugar	Cakes, biscuits and margarine.
14	Sugar	Refined sugar
15	Water	Mineral water

The number of calories that an average person needs on a daily basis is set based on the minimum (MDER) and average dietary energy requirements (ADER). However, specifically for this study, ADER was used since this indicator reflects the way in which FAO calculates the reference for adequate nutrition in the population. This value, according to FAO, was fixed for Peru at 2250 kcal per person and day in 2009 [40], a similar period of time to that of the diet patterns described above. However, as can be observed in Table 3, very few cities and other groupings that were analyzed reached this threshold only with the food that is purchased in the household. The reason for this lower registered value is linked to the amount of food that Peruvians consume as FAFH, which accounts on average for approximately 15% of the caloric intake [41], as explained below.

**Table 3:** Average daily dietary energy consumption per capita based on household purchase of food and out of household expenditure (food-away-from-home—FAFH) in different Peruvian cities.

Scenario	Household caloric intake	Away-from-home caloric intake	Total caloric intake	Caloric deficit^a^
	kcal per person per day			
*Average Peru*	*1899*	*252*	***2151***	*99*
Lima Metropolitana	1834	371	**2205**	45
Abancay	2101	400	**2501**	—
Arequipa	1778	317	**2095**	155
Ayacucho	1863	291	**2154**	96
Cajamarca	2591	355	**2946**	—
Chachapoyas	2162	366	**2528**	—
Chiclayo	1949	245	**2194**	56
Chimbote	2159	389	**2548**	—
Cusco	2128	513	**2641**	—
Huancavelica	2005	301	**2306**	—
Huancayo	2021	366	**2387**	—
Huánuco	1967	360	**2327**	—
Huaraz	2234	409	**2643**	—
Ica	2048	334	**2382**	—
Moquegua	1999	416	**2415**	—
Pasco	2013	282	**2296**	—
Piura	1891	282	**2173**	77
Pucallpa	1906	345	**2251**	—
Puerto Maldonado	2061	662	**2723**	—
Puno	1909	371	**2280**	—
Tacna	1832	435	**2267**	—
Tarapoto	1905	447	**2352**	—
Trujillo	2023	395	**2418**	—
Tumbes	1991	414	**2405**	—

^a^ Shortage in the amount of calories consumed relative to the amount of calories required for maintenance of current body weight. The amount of calories required was fixed based on the average dietary energy requirements (ADER) considered by the Food and Agriculture Organization of the United Nations (FAO) for Peru: 2250 kcal per capita per day (FAO, 2011).

#### Accounting for food loss throughout the supply chain

Food loss occurs throughout the supply chain in the pre-wholesaler stage. Waste, in contrast, occurs in a subsequent stage and is related to the consumer’s behavior [42]. Data reported in the ENAPREF survey represent the average per capita acquisition of food products in Peruvian households [36]. Therefore, these data do not represent the actual consumption of food, but the quantity that is purchased, not accounting for food waste in the household. Food loss was, therefore, accounted for in the assessment by following the regional estimations in the FAO report on food losses and waste [42]. Average losses for Latin America were extracted from this report as shown in Table 4.

**Table 4:** Food waste percentages per food commodity group in different stages of the supply chain in Latin America. Adapted from Gustavsson et al. (2011).

Food commodity	Agricultural production	Postharvest handling and storage	Processing and packaging	Distribution
Cereals	6%	4%	2%	4%
Roots and tubers	14%	14%	12%	3%
Oilseeds and pulses	6%	3%	8%	2%
Fruits and vegetables	20%	10%	20%	12%
Meat	5.3%	1.1%	5%	5%
Fish and seafood	5.7%	5%	9%	10%
Milk	3.5%	6%	2%	8%

#### Accounting for food-away-from-home (FAFH)

The ENAPREF survey conducted in 2008–2009 constitutes the basis on which to calculate inflation of food products in Peru. Unfortunately, information provided by the survey only details values for specific food products and categories purchased for the household, whereas the FAFH data only considers types of meals. Hence, FAFH was computed using the expenditure disaggregation of the consumer price index (also known as commodity bundle).

The procedure consisted in calculating the ratio between food purchase for the household and FAFH based on the latest consumer price index breakdown available (i.e., year 2014), as shown in Section—A1 in S1 File [41]. Thereafter, the price of the food basket purchased for the household was quantified in monetary units using the average price per food product from January 2016 to July 2016. Once this value was computed, the monetary value of FAFH was calculated.

To estimate the final amount of calories that an average person consumes away from home, the following procedure was undertaken. In the first place, the Peruvian Tables of Food Composition were used to calculate the total amount of kilocalories consumed by an average person in the household in each scenario. Once these were computed, the ratio between the average amount of kilocalories and household expenditure per person was obtained (kilocalories per Peruvian sol—PEN). In parallel, it must be noted that one monetary unit in the household is not equivalent to that of restaurants or canteens outside the household, since the structural costs differ considerably. Peruvian specialists were consulted to determine a coefficient that relates food costs with the final price paid by a customer in an average restaurant, which was set at 0.45, with the remaining 0.55 linked to paying personnel, VAT, general expenditures at the eatery and net profit (D’Gallia staff, Department of Culinary Economics, personal communication, February 2017). This coefficient was then multiplied by the FAFH economic expenditure and the caloric-monetary ratio in order to estimate the number of kilocalories consumed outside the household and, thereafter, the GHG emissions considering a fixed value of g CO_2_eq per kilocalorie in each scenario (obtained from household purchase). This fixed criterion was assumed based on the lack of information related to the food patterns outside the household. The rationale behind this assumption, which is further analyzed in the discussion section, revolves around the basic idea that consumer patterns in and out of the household may have a similar structure.

Due to data limitations, the FAFH estimation procedure was only replicated for the geographical and socioeconomic scenarios, although for the aggregated values of Coastal, Andean and Amazonian Peru the average expenditures used represented mean Peruvian values. Moreover, the socioeconomic scenarios assume that the ratio between food purchased in the household and FAFH was constant throughout the expenditure quintiles. Out of household expenditures and, therefore, derived nutritional and environmental estimations, were not computed for the academic status scenarios.

### Embodied GHG emissions in food products

Environmental data were available for a total of 68 food products, representing over 95% of the total basket in ENAPREF [36]. The excluded products are linked to spices and aggregated small quantities of “other” staples or cereals that could not be assigned to a specific item. However, it should be noted that certain goods, such as salt, pepper and other condiments are not included in the data reported by ENAPREF. Similarly, alcoholic beverages, such as beer or *pisco*, are not considered for computing purposes. Interestingly, a final group of products, including green asparagus, pomegranate or blueberries, correspond to products that have started to be produced on a large scale in Peru in the past few years, but are essentially destined to export to other continents. Therefore, these are not considered by the ENAPREF survey and discarded for the study.

GHG emission estimations followed standard LCA guidelines [43]. The impact category selected to compute these emissions was the Global Warming Potential (GWP) indicator [44]. This category is considered a midpoint, since it represents the direct emissions of GHGs that occur in the life cycle of a product, in other words, an intermediate point along the environmental mechanism that links anthropogenic interventions to a set of areas of protection (e.g., human health or ecosystem damage). GWP is calculated based on the radiative efficiency of each substance (i.e., GHG) and the time horizon over which the calculation is considered. In this particular study, a 100 year timeframe was selected, a consensus subjective value that is commonly used in LCA studies following the hierarchist perspective of the Cultural Theory [45]. Thereafter, CO_2_ equivalency factors for all the inventoried GHGs were used as shown in A1 Table in S2 File in order to report the final GWP value through the consensus unit of reference: kilograms of CO_2_eq.

Although several studies using life-cycle methods have been developed in Peru in recent years, these were not sufficient to fulfil the complexity of the entire basket of products that were considered in the study. Nevertheless, when available, GHG emissions from local LCA or Carbon Footprint studies were used [33–34, 46–47]. Otherwise, data from two main sources were used to obtain the GHG emissions. On the one hand, GHG emissions results extracted from the ecoinvent^®^ v3.2 database were used for a series of vegetable and fruit products, including papaya, celery, cabbage, lettuce or grapes. On the other hand, most of the data for the remaining food products included in Table 5, for which no GHG emissions were available either in a Peruvian context or in the ecoinvent^®^ v3.2 database, were extracted from a review performed by Clune et al. [48] for 168 different food products from over 350 scientific publications. The environmental impact of banana production was modelled based on the study developed in Ecuador by Roibás et al. [49]. Table 5 presents the list of food products included in the assessment with their average GHG emissions value (Uncertainty for the reported values can be found in Section A4 in S1 File).

**Table 5:** Individual Global Warming Potential (GWP) values (kg CO_2_eq/kg of produce or bone free meat) for the different food products considered.

Product name	Food Category	Average GWP value (kg CO_2_eq/kg of produce or bone free meat)	Bibliographical sources for GWP
Rice	1	0.913	Quispe et al. (unpublished)
Maize	1	0.656	Clune et al. 2017
Wheat	1	0.934	ecoinvent 3.2
Oats	1	0.873	ecoinvent 3.2
Barley	1	0.748	ecoinvent 3.2
Bread	1	1.600	Kulak et al. 2015
Flour (wheat)	2	1.016	ecoinvent 3.2
Flour (legumes)	2	1.030	ecoinvent 3.2
Pasta	2	0.810	Wallén et al. 2004
Sweet potato	2	0.356	Clune et al. 2017
Potato	2	0.356	Clune et al. 2017
Yuca	2	0.356	Clune et al. 2017
*Olluco*	2	0.356	Clune et al. 2017
*Chuño*	2	0.356	Clune et al. 2017
Celery	3	0.572	ecoinvent 3.2
Lettuce	3	0.321	ecoinvent 3.2
Coles	3	0.476	Clune et al. 2017
Peppers	3	0.996	Clune et al. 2017
Tomato	3	1.566	ecoinvent 3.2
*Cucurbita* spp.	3	0.332	ecoinvent 3.2
Peruvian corn (*choclo*)	3	0.716	ecoinvent 3.2
*Calabaza*	3	0.332	ecoinvent 3.2
Garlic	3	0.575	Clune et al. 2017
Onion	3	0.567	ecoinvent 3.2
Carrot	3	0.220	Clune et al. 2017
Lemon	4	0.216	Clune et al. 2017
Mandarin orange	4	0.712	Bartl et al. 2012
Orange	4	0.266	Clune et al. 2017
Peach	4	0.747	Bartl et al. 2012
Apple	4	0.537	Bartl et al. 2012
Avocado	4	0.656	Bartl et al. 2012
Papaya	4	0.293	ecoinvent 3.2
Banana	4	0.300	Roibás et al. 2016
Grape	4	0.367	ecoinvent 3.2
Strawberry	4	0.480	Bartl et al. 2012
Mango	4	0.295	Basset-Mens et al. 2014
Watermelon	4	1.556	Mohammadi et al. 2016
Vegetable oil (L)	5	4.140	Wallén et al. 2004
Fresh milk (L)	6	1.690	Clune et al. 2017
Pasteurized milk	6	1.690	Clune et al. 2017
Evaporated milk	6	1.690	Clune et al. 2017
Yoghurt	6	1.300	Clune et al. 2017
Fresh cheese	6	8.730	Clune et al. 2017
Fish (ocean)	7	0.115	Avadí (2014)
Fish (river)	7	1.940	Avadí (2014)
Dried or salted fish and shellfish	7	0.425	Avadí (2014)
Canned fish and shellfish	7	1.728	Avadí (2014)
Other poultry	8	5.910	Clune et al. 2017
Hen	8	3.990	Clune et al. 2017
Chicken	8	3.990	Clune et al. 2017
Poultry	8	5.910	Clune et al. 2017
Eggs	8	3.260	Clune et al. 2017
Common beans	9	0.510	Clune et al. 2017
Peas (dried or fresh)	9	0.600	Clune et al. 2017
Fava beans	9	0.510	Clune et al. 2017
Lentils	9	1.030	Clune et al. 2017
Lamb	11	27.910	Clune et al. 2017
Pork	11	5.720	Clune et al. 2017
Beef	11	28.600	Clune et al. 2017
Offal	11	28.600	Clune et al. 2017
Ice cream	12	0.640	Wallén et al. 2004
Soft drink	13	0.560	Wallén et al. 2004
Flavored juice	13	0.560	Wallén et al. 2004
Biscuits	14	2.640	Wallén et al. 2004
Cakes and other	14	2.640	Wallén et al. 2004
Margarine	14	2.120	Wallén et al. 2004
Sugar	15	0.480	ecoinvent 3.2
Bottled mineral water	16	0.079	Garfi et al. 2016

The system boundary assumed for each product corresponds to the cultivation stage in the case of agricultural and aquaculture products, the fishing and landing of seafood and grazing and slaughter houses in the case of livestock. In all cases, the supply chain was followed until each food product was at the gate of the farm, port or processing plant ready for distribution to wholesalers. Transportation to regional distribution centers (RDCs) was only included for those scenarios for which it was feasible to do so, as shown in Table 6. In this sense, the functional unit (FU) used was 1 kg of product at an RDC ready for retailing, except in the case of milk and other liquid products, such as juices, oils or bottled water, where the FU was 1 L of liquid content. In the case of meat products the FU can be very variable. For this study, a bone free meat FU was assumed for red meat and poultry. In those scenarios in which distribution was not included, the FU was limited 1 kg of product at the gate of the farm, port or processing plant ready for distribution to an RDC.

**Table 6:** System boundary of the production system analyzed.

Scenarios	Production	Processing	Distribution to regional distribution center
***Geograhical***			
• National average	✓	✓	-
• Cities	✓	✓	✓
***Socioeconomic***			
• Lima	✓	✓	✓
• Regional (coast, Andes and Amazon)	✓	✓	-
***Educational***			
• National average	✓	✓	-

#### Regional distribution of food products

Final distribution of food products to an RDC was modelled based on the production zones. The distance that a particular food product covers from its production site up to the final consumer is named “food miles”. Databases from different Peruvian institutions were used to obtain agriculture and cattle production information [50]. From these databases, the annual production of each food product was extracted and disaggregated per region. Thereafter, a selection of the main production sites per product was performed, using 2.5% of national production per region as the cut-off criterion to recalculate a new weighted production rate. This allowed accounting for at least 75% of the national production in all the food products included in the study. This criterion was used due to the fact that the majority of the production is located in a small number of regions (usually four or five) throughout the country, whereas the remaining regions have minimal production rates that do not even fulfil local needs (see Section A2 in S1 File). The rationale behind this assumption is that the new estimated production rates remain fixed across the different cities, assuming that the distribution of food is carried out homogeneously across the nation. What changes, therefore, is the amount of each food type purchased (and consumed) in each city, as well as the distance from the producing regions to the city in which the food is purchased.

To calculate the distance from a site of production to each of the cities evaluated, a plugin in QGIS was used [51]. Considering that cultivation sites are scattered throughout the Peruvian geography, it was assumed that the freight originates in the centroid of each production region and is transported up to the center of the city under assessment (see A3 Table in S1 File for the computed distances). However, it should be noted that if the centroid of the region did not coincide with the existence of a road, the centroid was adjusted to the closest road included in the database. These distances were then multiplied by the number of metric tons produced per food product in each region to obtain new values in metric tons per kilometer. Thereafter, using the ecoinvent^®^ v3.2 database, CO_2_eq emissions were computed for the different freight types: conventional freight and freight with refrigeration. This process was repeated for every single product and for each city included in the assessment (details available in S1 File).

As an example, mango is produced mainly in the North coast in the regions of Piura (73.4%), Lambayeque (12.5%) and Cajamarca (2.6%). The cut-off criterion is satisfied, as all three regions contribute at least 2.5% to the national production. Once a new weighted production rate is estimated exclusively based on these three regions, three new factors, 0.83, 0.14 and 0.03, respectively, are obtained. If mangoes are be freighted to *Lima Metropolitana*, were an average citizen consumes 2.1 kg per year, the distance from the centroid of these two regions is 1046 km in the case of Piura, 862 km for Lambayeque and 985 km for Cajamarca. Hence, a final tkm value of 2.43 is calculated for the road freight of 1 metric ton of mango to the capital city. Considering a factor of 0.168 kg CO_2_eq per tkm to transport a non-refrigerated product, a final distribution emission of 0.41 kg CO_2_eq of mango was estimated for this example.

Distribution emissions were not computed for the academic status scenarios and for the average GHG emissions for Peru due to the difficulty of locating an exact destination point when using these aggregated values. Similarly, for the socioeconomic scenarios, only distances linked to the expenditure quintiles for the city of Lima were modelled (see Table 6).

#### Lump sum

**Fig 1:**
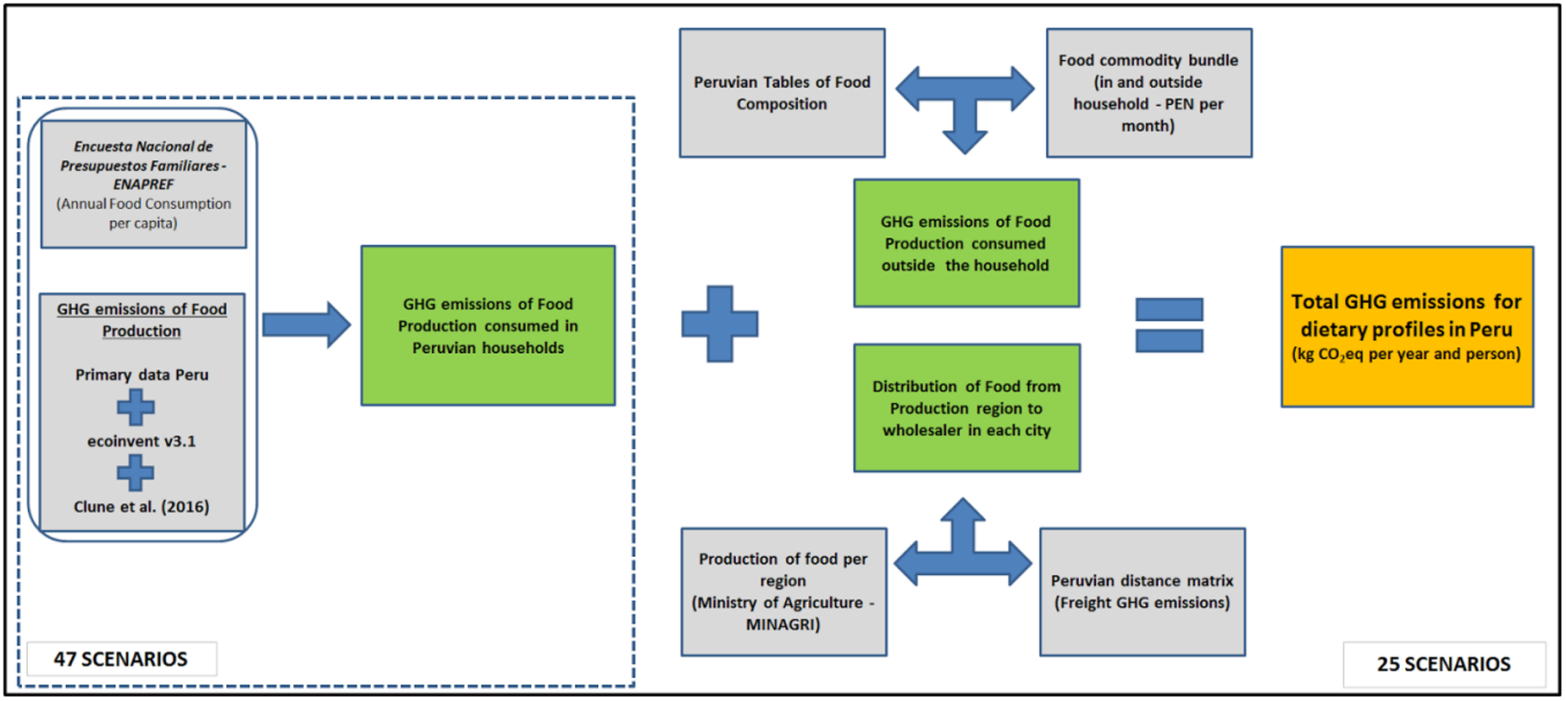
Schematic representation of the model created to estimate greenhouse gas (GHG) emissions linked to dietary patterns in Peru. Grey boxes represent raw data processing, green boxes partial results and the orange box represents the final GHG emissions per scenario.

Once the GHG emissions from the production of food included in ENAPREF were computed for each city, these were added to the estimation of GHG emissions related to FAFH and those linked to the regional distribution of food up to the city of consumption, as shown in Fig 1. In total 25 geographical scenarios were computed with all the stages. The scenario for the Peruvian average diet only included the quantification of production of food, without considering regional distribution to the wholesaler. Finally, socioeconomic scenarios only included the production of food consumed in the household.

### Assumptions and limitations

The city of Iquitos was excluded from the assessment due to the lack of road transportation of products to this city. This decision is linked to the fact that, although air freighting tends to generate much higher GHG emissions than road, train or marine freight, the construction of a new road network to the city of Iquitos through the Amazon rainforest would probably trigger important environmental impacts, not only in terms of GHG emissions, but also regarding loss of biodiversity [52–53].

Despite the fact that Peru is an agricultural nation and is self-sufficient for most of the main food items that are consumed domestically, this is not extendable to all. An extreme case is wheat, for which imports represent approximately 85% of the total consumption [54]. Imports of maize, barley or apples are also relevant to supply domestic demand [55]. In contrast, over 90% of rice is produced within Peru, and the only potatoes that are imported are those which supply fast food restaurants [56]. Modelling in the study considered that food production in Peru was all domestically produced. An important motive for doing so was the fact that the origin of imported food stock was not traceable through publicly available data with the level of details required. Nevertheless, these limitations were considered when conducting the uncertainty analysis described in the following subsection, by penalizing those food products with poor data quality in terms of geographical origin.

Direct land use changes were included within the system boundary of the food products selected. However, it should be noted that these, considering the fact that most GHG emissions are not actual data measurements for Peru, are not site-specific for domestic conditions. In contrast, indirect land use changes were not considered within the scope of the study.

### Uncertainty analysis

Uncertainties when dealing with large samples and aggregated data can be substantial. Hence, the computation of uncertainty can help understand the meaningfulness of the results. The data sources used to construct the GHG emissions per food product in this study demonstrate that data availability is an important obstacle when aiming at assessing diet-related environmental impacts [45, 57]. Therefore, it is important to provide readers with information regarding variability within sample through the use of standard deviations [58]. Consequently, standard deviations for the GHG emissions for each individual product have been included in the study, with the aim of providing upper and lower estimations of carbon footprint for each individual food product.

Given the heterogeneous origin of the LCI entries included in the study, the Pedigree matrix was used to calculate the underlying stochastic distributions in a uniform manner for all the GHG data values [59–60]. This approach enabled coding qualitative expert judgement by the LCA practitioners involved in the current study (see Section A2 in S2 File for details) into a numerical scale assuming that each stochastic inventory follows a lognormal distribution [61]. In this sense, data sources were assessed based on five different quality indicators (i.e., reliability, completeness, temporal correlation, geographical correlation and further technological correlation). Consequently, the nominal value of the GHG emissions per food product is considered the geometric mean of the distribution, whereas the geometric standard deviation is computed by using the aforementioned Pedigree matrix, as shown in Eq 1:

$$\sigma_{g}^{2}=\text{exp}\sqrt{{\left(\text{ln}U_{1}\right)}^{2}+{\left(\text{ln}U_{2}\right)}^{2}+{\left(\text{ln}U_{3}\right)}^{2}+{\left(\text{ln}U_{4}\right)}^{2}+{{\left(\text{ln}U_{5}\right)}^{2}+{\left(\text{ln}U_{6}\right)}^{2}+(\text{ln}U_{b})}^{2}}$$ (1)

Where U_1_ represents reliability, U2 completeness, U3 temporal correlation, U4 geographical correlation, U5 further technological correlation and U_b_ corresponds to an uncertainty factor which is linked to the environmental burden under assessment. This equation enables the calculation of two uncertainty levels (low and high), that are used to compute the standard deviation of the GHG emissions of each individual food product. Details on how the scores are considered in the Pedigree matrix are described in Section A2 in S2 File (see A2, A3 and A4 Tables), whereas the calculation of the U_b_ parameter is shown in Section A4 in S1 File.

## Results

### GHG emissions for the average Peruvian diet

**Fig 2:**
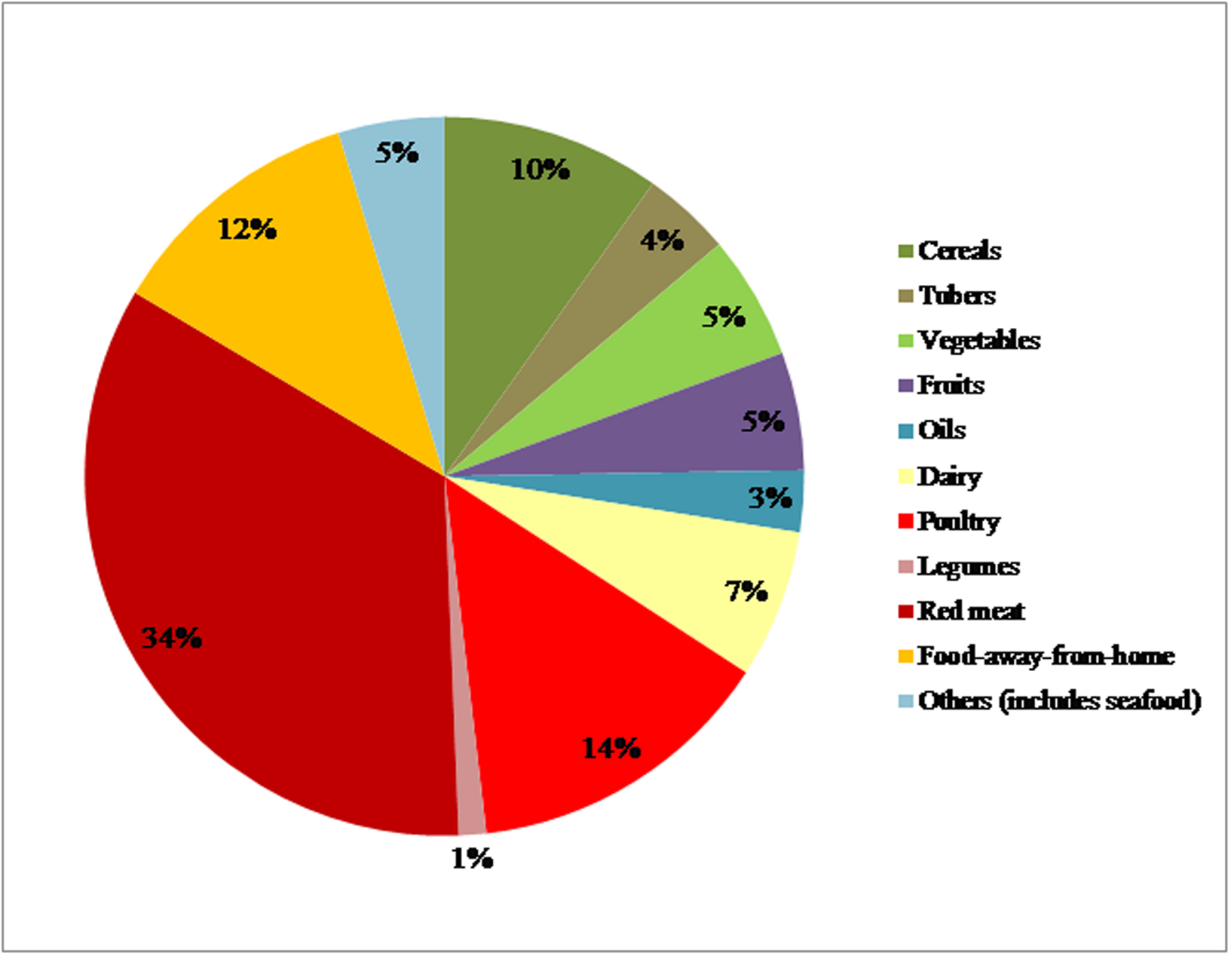
Relative contribution of GHG emissions from food production per food category to the average Peruvian diet. Results include food-away-from-home (FAFH), but exclude distribution to a regional distribution center.

GHG emissions linked to production of food purchased for the household represented 956 kg CO_2_eq per year per capita for the average conditions of Peru. The main food category that contributed to this amount was red meat, representing 39% (369 kg CO_2_eq), followed by poultry, 16% (151 kg CO_2_eq), cereals, 11% (107 kg CO_2_eq), and vegetables, 6.3% (60.5 kg CO_2_eq). When the GHG emissions linked to FAFH production are added, the total footprint increases to 1.08 t CO_2_eq. Therefore, FAFH would represent approximately 12% of total GHG emissions from a food production perspective. Distribution was excluded from this scenario (see Fig 2).

### GHG emissions per city (geographical scenarios)

**Fig 3:**
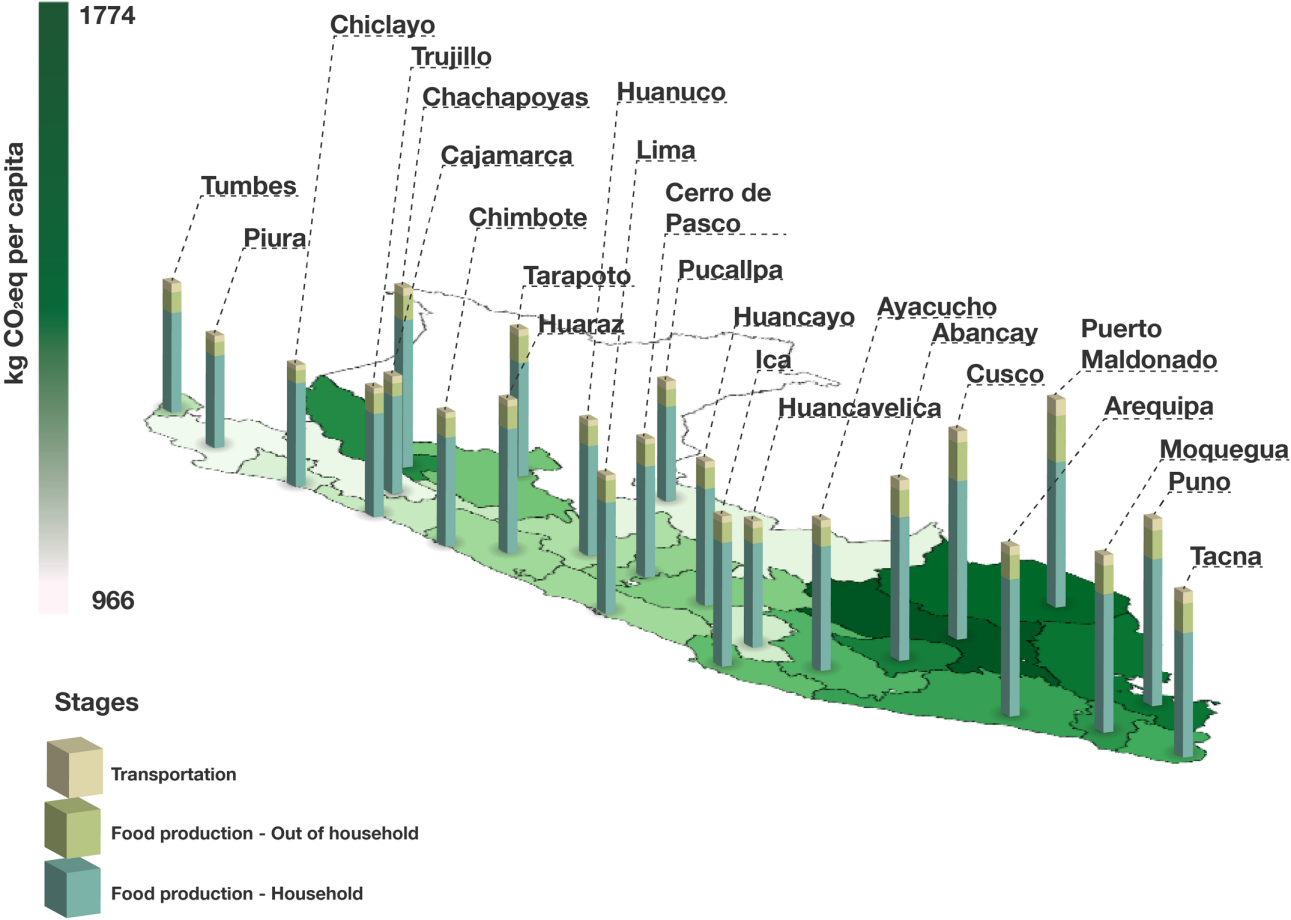
Annual dietary GHG emissions per capita based on city. Results include food-away-from-home (FAFH) and distribution to regional distribution centers.

The average GHG emissions for total food production in each of the cities assessed ranged from 966 kg CO_2_eq in the city of Piura to 1.77 t in Cusco (see Fig 3). Cusco is located in the Andes, which was the area for which the average city showed the highest GHG emissions per capita (1.36 t CO_2_eq), whereas the average coastal city presented a value of 1.20 t CO_2_eq and the three cities analyzed in the Amazon basin showed an average value of 1.35 t CO_2_eq. However, it should be noted that whereas cities along the Peruvian coast showed a lower range of values, these were more significant in the Andes and the Amazon basin. For instance, in the case of the Amazon basin, the city of Puerto Maldonado presented an average footprint of 1.77 t CO_2_eq, whereas in Pucallpa the average value was 1.03 t CO_2_eq.

Although the range of values may appear to be rather limited, important differences between cities can be identified when observing the relative contribution of each of the 15 food type groups that have been used. Notably, red meat products, represent more than 40% of the total impact in the cities of Chachapoyas (44%), Arequipa (41%), Cusco and Puno (40%), all Andean cities, whereas the lowest proportion of red meat-related emissions was observed in Cajamarca (Andes, 20%) and Pucallpa (Amazon, 25%). In addition, if all sources of animal protein are aggregated, these represent between 35% (Cajamarca) and 57% (Chachapoyas) of the total impact, although every city except Cajamarca presented values above 45%. Production of food consumed beyond the household ranged from 11% (112 kg CO_2_eq) in Chiclayo (northern coast) to 22% in the city of Puerto Maldonado (398 kg CO_2_eq). GHG emissions linked to product distribution to wholesalers were most noticeable in the cities of Puerto Maldonado and Pucallpa, in the Peruvian Amazon, and in the cities of Tumbes and Tacna along the Peruvian coast. These four cities have in common two key issues that influence this high value: a) low food production rates in their region; and, b) increased road distances to other areas within the country.

When the average contribution of each food category is analyzed per natural area, coastal and Amazon cities show a considerably lower proportion of environmental impacts linked to the consumption of red meat (30% and 30%, respectively) as compared to Andean cities (35%). However, this difference is compensated, to a certain extent, by higher relative environmental impacts linked to poultry consumption in the former areas. It is interesting to note that substantial differences between areas are visible in terms of GHG emissions linked to the following food types: red meat, seafood, dairy products, cereals and poultry, suggesting important social differences in terms of food intake in the three natural areas.

*Lima Metropolitana* shows a total GHG emissions value of 1.18 t CO_2_eq, situating the capital city among the cities with the lowest associated environmental impacts. Although the relative proportion of most food types in Lima in most cases did not show relevant differences to the average in cities, poultry (14%) and FAFH (16%) were above the average Peruvian city. In addition, the proportion of GHG emissions linked to products rich in sugar (e.g., ice cream or soft drinks) was one of the highest in the country (4.0%). In contrast, impacts linked to distribution represented 3.7% of the total impact, the lowest value for all cities due to the central geographical location of *Lima Metropolitana*, as well as to the radial road network existent in Peru.

### GHG emissions based on socioeconomic scenarios

**Fig 4:**
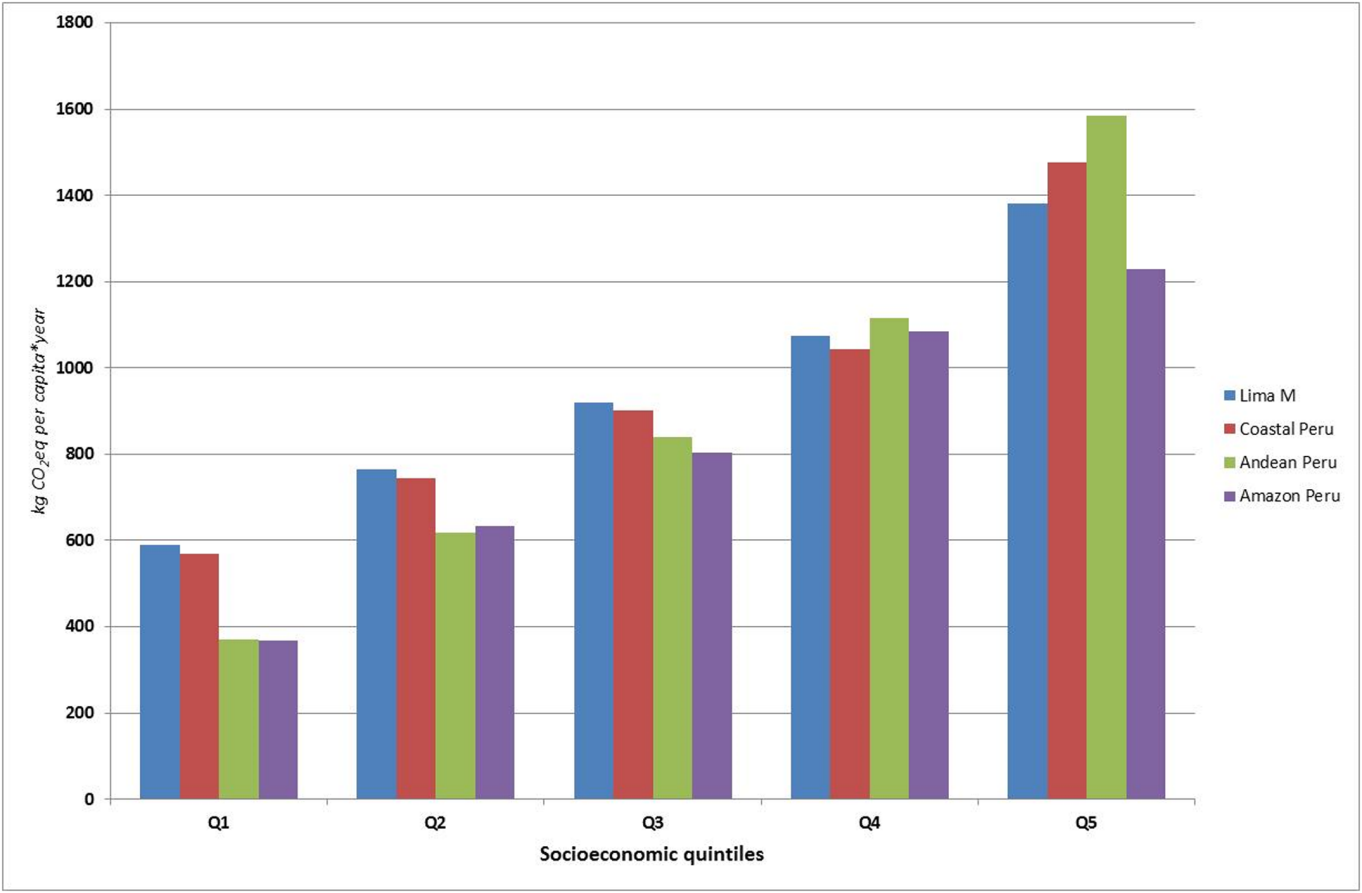
Annual dietary GHG emissions per capita based on socioeconomic quintiles in each natural area of Peru and in *Lima Metropolitana*. Results exclude GHG emissions linked to distribution of food products to regional distribution centers. Results for Coastal Peru include *Lima Metropolitana*.

The GHG emissions related to the production of food purchased for household per capita presented relevant variations when analyzed from a socioeconomic perspective. For instance, for *Lima Metropolitana* the GHG emissions per economic income shows a very strong correlation between higher economic standards and higher GHG emissions associated with the average diet, as shown in Fig 4. In fact, GHG emissions for quintile 5 (the wealthiest group) are 134% higher than for the poorest quintile. Similar trends are observable when urban environments are analyzed based on quintiles in other coastal cities. However, when Andean and Amazon cities are examined, the disparity between Q1 and Q5 increases substantially (326% for the former and 234% for the latter). Hence, in the case of Andean cities GHG emissions in Q1 added up to 372 kg CO_2_eq, whereas in the case of Q5 this value rose to 1.58 t CO_2_eq.

When the results are presented in terms of the educational level of the head of the family, a significant difference is also visible between the GHG emissions in households where the family head has no academic degree (841 kg CO_2_eq) and households in which the family head has such academic qualification (1.17 t CO_2_eq).

### Uncertainty analysis

Uncertainty analysis was conducted for the production of food consumed in the household. The results, which can be observed in Section A4 in S1 File, were computed to strengthen the robustness of the study and its applicability in policy support. As shown, the geometric variance was highest for mango, barley and flour (1.28), due to the higher uncertainty linked to geographical and temporal correlation. Hence, considering that these products show the highest standard deviations, and their carbon footprint is not calculated based on a Peruvian sample, it seems plausible that future LCA research in the food industry in Peru should include them. In contrast, products with a carbon footprint which has been calculated based on recent studies conducted in Peru, such as rice (1.04), and to a lesser extent fish products (1.10) and fresh milk (1.11), presented the lowest geometric variance. Lamb (1.16), pork, and beef (1.14) presented higher geometric variance than that of fish products. In a similar line to those products with the highest geometrical variance, red meat products, which were not calculated specifically for Peru, but rather for South America, merit future analysis considering the high GHG emissions related to them.

## Discussion

### Geographical variations in GHGs emission of dietary patterns

As mentioned in section 3.2, certain differences in terms of GHG emissions can be observed between the main cities in Peru, and a certain tendency can be identified regarding the fact that cities in the Andes present higher environmental impacts. This leads to the obvious inference that differences in environmental impact may be due to two main issues: i) different patterns of consumption regarding the types and groups of food products purchased; and, ii) different expenditure levels of food in and outside the household. For the former, Fig 3 is clear in showing that cities in the Andes, for instance, tend to consume a higher proportion of animal protein, mainly beef and pork, whereas coastal and Amazon basin cities show a lower consumption of animal protein. Moreover, in coastal and Amazon basin areas a higher proportion of animal protein intake is obtained from consumption of fish, which tends to have lower GHG emissions than beef or pork per mass unit [62].

Based on the data shown in Section A1 in S1 File, expenditure levels linked to FAFH consumption are highest in cities in which household consumption also presents the highest GHG emissions (i.e., Cusco and Puerto Maldonado), in line with studies reported elsewhere [63]. However, it should be noted that accounting for FAFH GHG emissions is based on the same distribution of food intake as in the household due to lack of statistical data for behavior regarding meals outside the household. While we consider that this is a reasonable proxy to present a complete computation of diet-related GHG emissions, it is important to consider that several studies in the literature have examined the per capita expenditure composition of FAFH food. For instance, Ma et al. [63] identified that in 1998 Chinese households were spending 38% of FAFH total expenditure on meats, whereas expenditure on meats in the household was only 28%. Similar trends were identified for seafood products, whereas inverse tendencies were found for grains, fruits and eggs. In addition, important differences have been identified in FAFH depending on the time of day, on the type of meal, on socioeconomic classes and on whether the expenditure is linked to leisure time or necessity [64–65].

When linking the results obtained in terms of geographical variation with those regarding the incidence of non-communicable diseases (NCDs) in different areas of Peru based on official national data [21], no clear tendencies were visible. Interestingly, the city with highest GHG emissions per capita in the diet also presented the highest rate of people overweight in the nation: Puerto Maldonado. However, no clear relationships were seen between the proportion of overweight people and the GHG emissions when all the cities were examined.

### Effects of socioeconomic factors on GHGs emission of dietary patterns

In contrast to what has been observed on a geographical basis, differences between socioeconomic quintiles are very evident and significant from a final GHG emissions value perspective. For instance, in the case of *Lima Metropolitana* the GHG emissions of Q1 were 590 kg CO_2_eq, whereas Q5 shows values above 1.38 t CO_2_eq. It should be noted, however, that these disparities are due, not only to differences in food consumption patterns, but also to differences in caloric intake. Nevertheless, if the GHG emissions per kcal are quantified, there is an increase from 1.18 g CO_2_eq in quintile 1 to 1.62 g CO_2_eq in quintile 5. The main reason for this difference is the fact that, for instance, the consumption of beef in the Peruvian capital is over seven times higher for quintile 5 (11.3 kg/year) as compared to quintile 1 (1.5 kg/year). Similar tendencies were found in a study conducted by Wilson et al. [66] in New Zealand aiming at modeling several diet patterns based on health, cost and sustainability.

Several studies suggest that FAFH expenditure is highly dependent on socioeconomic groups, with wealthy households spending substantially more money on food in restaurants [64]. Furthermore, Ma et al. [63] show that Chinese households with the highest income spend 38% of their FAFH on meat products (51% if seafood is included) over a total of 1100 monetary units (i.e., Chinese yuan), whereas the lowest income households spend only 21% of FAFH expenditures on meats (27% including seafood) over a total of 164 monetary units. Consequently, although FAFH- and distribution-related GHG emissions were not included in the socioeconomic scenarios, we hypothesize that the inclusion of FAFH expenditure would further expand the gaps between the different income groups.

Nevertheless, it is plausible to assume that current consumption patterns in low income socioeconomic groups are bound to have improved in terms of caloric intake ever since the survey was conducted in 2008–2009, considering that GDP per capita and purchase power has grown considerably, together with a significant reduction in poverty and extreme poverty rates [67]. The distribution of food consumption in these social groups shows that access to certain food products, mainly animal protein and fruit, is an important challenge from a nutritional and health perspective. However, from an environmental approach these consumption patterns should be seen as having promising characteristics, which if managed correctly through policy actions, could mitigate the increase in red meat consumption and other high carbon products that is expected when purchase power increases.

### Nutrition and GHG emissions

Following the USDA [9] nutritional approach based on caloric intake, instead of the traditional number of servings approach, the Peruvian average protein intake diet is 20% lower than the recommended diet. Similar patterns are found for other food groups such as fruit and vegetables, 70% and 40% lower respectively. Although certain trends can be explained due to cultural reasons, since traditionally the Peruvian diet consists in large amounts of grains/cereals (rice, maize, wheat…), most of the average intake shows worrying results [2]. For instance, it should be noted that caloric intake from oils, fats and sweets accounts for 23% of the total. In fact, the NCD survey conducted in 2014 in Peru shows that only 10.8% of the adult population consumes 5 or more portions of fruit or vegetables per day, a value that is as low as 9.8% in the Amazon basin and 5.3% in the highlands [21]. Although optimizing Peruvian food dietary patterns is the main objective of a companion paper in the frame of the same research project and is consequently not analyzed in detail in the current study, we argue that adequate policy-making in Peru to improve the healthiness of the average diet should be analyzed together with efforts to mitigate GHG emissions and other environmental impacts.

### Contextualizing the results on a global level

When the results are contextualized and benchmarked to other diet-related GHG emissions studies, these comparisons, as in any other LCA study, should be interpreted with caution due to the wide array of methodological approaches, system boundaries or FUs considered [68]. Table 7 depicts the diet-related GHG emissions for other studies that assumed similar methodological assumptions. Thus, the average Peruvian diet is in the lower range of GHG emissions in comparison to developed countries. Interestingly, results for Peru are aligned to those obtained for vegan or vegetarian diets, which are usually reported as being more sustainable in terms of GHG emissions and other environmental impacts [68], in spite of having similar animal based protein intake to other diet-related studies [13, 17, 69]. Nevertheless, it should be noted that the average caloric intake in most of the nations considered in Table 7 is far above 2500 kcal/day, whereas in Peruvian cities this value ranges from approximately 2100–2700 kcal/day [35].

**Table 7:** Annual dietary GHG emissions per capita as compared to other results in the bibliography. Results reported per kg CO_2_eq/person year.

Source	Country	Diet type/Diet location	kg CO_2_eq/person year
Current study	Peru	Average Peruvian	951^a^
		Cusco	1774^b^
		Piura	966^b^
Vieux et al. (2013) [17]	France	Average sample	1522
		Men	1724
		Women	1338
Werner et al. (2013) [69]	Denmark	Average dairy	1690
		High dairy	1650
		Non-dairy	1695
		Soy drink	1321
		Vegetarian	1118
		Vegan	881
Scarborough et al. (2014) [13]	UK	High meat-eaters (>100 g/day)	2624
		Medium meat-eaters (50–99 g/day)	2055
		Low meat-eaters (<50 g/day)	1705
		Fish eaters	1427
		Vegetarians	1391
		Vegans	1055
Wilson et al. (2013) [66]	New Zealand	Low cost	876
		Low cost and low GHG emissions	595
		NZ-Pacific theme	2183
		NZ average	3687
Pairotti et al. (2017) [70]	Italy	Italian average	2010
		Vegetarian	1750
		Mediterranean	1870
Risku-Norja et al. (2009) [71]	Finland	Average Finnish	1700
		Healthy	1400
		Non-dairy, ruminant meat replaced by pork and poultry	1100
		Vegan	900
Muñoz et al. (2010) [72]	Spain	Average for Spain	2100

^a^ Excludes transport of food products to a regional distribution center.

^b^ Includes transport of food products to a regional distribution center.

Peruvian diets appear to be on the lower range of GHG emissions when compared to Western societies. However, the fact that residues in Peru are mainly disposed of in dumpsters, rather than in the very few (but increasing) landfills throughout the country, may imply a strong environmental impact towards the end-of-life of food products. For instance, a study conducted by Manfredi et al. [73] suggested that waste emissions in dumpsters can be as high as 1 t CO_2_eq per metric ton of waste, whereas in conventional landfills this value is reduced to roughly 300 kg CO_2_eq/t. Therefore, in this context, the current transition that has been implemented in Peru from informal dumpsters to landfills may translate into important GHG emission reductions that would mitigate the expected increase in the carbon footprint of the average diet as computed in the current study [74].

### GHG emission reductions in the Peruvian diet and policy actions

Quinoa, an emblematic Peruvian food product produced in the Andes at altitudes that range from 2800 m to 3600 m, has a minor role in the Peruvian diet, as can be observed in the data used in the current study. However, a parallel study has determined that the GHG emissions linked to quinoa during its production and distribution sum up to roughly 1.03 kg CO_2_eq per kg of quinoa. Considering its high protein content (13.6 grams per 100 grams of product), substantial GHG emission reductions could be attained if the Peruvian diet shifted to quinoa consumption while removing meat [39]. We acknowledge that this shift would imply important economic and social implications, especially because any environmentally sustainable improvement in diet quality in emerging and developing nations assumes the availability of nutritious foods at affordable prices, especially for poor socioeconomic groups [75]. However, we argue that from an environmental perspective it could have substantial benefits. For instance, if 20% of beef-based protein consumption in the average Peruvian diet were to be substituted by quinoa consumption, GHG emissions could be reduced by 30.9 kg CO_2_eq per capita each year, the equivalent of 973 kton CO_2_eq per year in all Peru.

Food replacement by more sustainable products must be done thoughtfully. In this sense, Werner et al. (2013) found that diet optimization regarding GHG emissions can lead to nutritional consequences when dairy products are excluded [69]. Similarly, Vieux et al. [17] found that when animal protein intake is replaced iso-calorically by fruits and vegetables, diet-related GHGs tend to augment. However, we argue that a potential expansion of the agricultural frontier to produce more quinoa would occur in the Peruvian highlands. In this area direct and indirect land use changes would translate into lower GHG emissions due to deforestation than in areas at lower altitudes, namely the Amazon basin, where there is a potential threat of deforestation because of cattle ranching [76–77]. Moreover, quinoa production still shows vast areas in which there is room for increasing yields considerably [78], something which constitutes a major challenge in many tropical nations [79].

Considering that Peru will have to comply with a 31% reduction in its GHG emissions by 2030 in the frame of the Paris Accord, it is surprising to see that none of the reductions included in the Nationally Determined Contributions (NDCs) proposed by the Peruvian government are linked to seeking changes in Peruvian dietary patterns. In fact, the only NDCs aimed at reducing GHG emissions in the agricultural sector sum up to approximately 1.45 Mt CO_2_eq per year, a minor contribution considering a total reduction of 82.2 Mt CO_2_eq per year that is projected for 2030 [26]. Hence, we hold that food production and distribution should play a more important role in the national strategy to reduce GHG emissions.

Results obtained from this study could help policy-makers to take action regarding legislation on food and diet optimization. Thus, low carbon and healthy food could be promoted in canteens, especially in educational centers (like schools, universities, etc.) together with other healthy food promotion schemes. Also, due to the excess of fat and oil calories intake, taxes for sodas, refined sugars or unhealthy-fatty snacks, such as those recently implemented in Canada, Denmark or Mexico, can be implemented within a broader context of “health related-food taxes” to dissuade people from an excessive consumption of these products [80–81]. However, these legislative restrictions should be handled with care in order to avoid unintended rebound-effects that could ultimately counterbalance the positive health impacts they seek [82]. In addition, food-labelling policies may be useful for consumers’ decision-making in the supermarket. Thus, labels providing information regarding environmental and health issues would assist consumers during the purchasing process [17].

Finally, food loss, which was included in the computational analysis, but scarcely discussed in the current study, should not be neglected from a policy perspective. Despite the complexity of specific interventions to mitigate food loss throughout the food supply chain, a 20% reduction of food losses throughout production, storage and processing would imply a reduction of 6.0% in the production of food for the average Peruvian diet (i.e., 897 kg CO_2_eq). If implemented on a national scale, the total reductions in GHG emissions considering the current Peruvian population would be 2.03 Mt CO_2_eq.

## Conclusions

As far as the authors were able to ascertain, this is the first study in which an environmental indicator has been estimated for average Peruvian dietary patterns. Results demonstrate that there are certain differences in terms of food intake from a geographical perspective, with distinct food consumption patterns on the northern coast, in the Andean highlands and in the Amazon basin. Considering GHG emissions from the production and distribution of food for household and FAFH consumption, the variation between the average citizens of the cities assessed ranged from 1.0 t to 1.8 t CO_2_eq.

Interestingly, from a socioeconomic point of view, there is a fivefold increase in GHG emissions between the lowest and highest expenditure quintiles for food consumption in the household. The carrying factor for this variation is the fact that lower classes have limited access to high carbon-emission food products such as red meat, and, to a lesser extent, a lower caloric intake per capita. In fact, unlike the current European or North American consumer, higher levels of obesity are found in Peruvian quintiles with higher purchase power.

The findings in this study demonstrated that actions in terms of food loss reduction or the substitution of ruminant meat in the diet for other, less carbon intensive, protein rich food products can lower per capita emissions considerably. In fact, some of these actions would imply substantial mitigations of GHG emissions on a national level. However, it should be noted that sustained economic growth and the expansion of the medium class in Peru could cause a substantial increase in the amount of animal protein consumed. When added to population growth and the risk of increased cattle ranching, this increase in animal protein consumption could mean Peru facing a struggle to comply with its international commitments in terms of climate change mitigation unless adequate policies are put in place.

## Supporting information

S1 File (XLSM)

S2 File (DOCX)
