# Supplementary material for: Climate change mitigation opportunities based on carbon footprint estimates of dietary patterns in Peru
Source: PLoS One. 2017 Nov 16;12(11):e0188182. doi: 10.1371/journal.pone.0188182 (PMC5690589; doi:10.1371/journal.pone.0188182)
Supplement: S2 File — (DOCX) [file pone.0188182.s002.docx]

**Climate change mitigation opportunities based on carbon footprint estimates of food dietary patterns in Peru**

Ian Vázquez-Rowe*^,1^, Gustavo Larrea-Gallegos^1^, Pedro Villanueva-Rey^1,2^, Alessandro Gilardino^1^

^1^ Peruvian LCA Network, Department of Engineering, Pontificia Universidad Católica del Perú, Avenida Universitaria 1801, San Miguel 15088, Lima, Peru.

^2^ Department of Chemical Engineering, Institute of Technology, Universidade de Santiago de Compostela, 15782 Santiago de Compostela, Galicia, Spain

*Corresponding author: Ian Vázquez-Rowe; e-mail: ian.vazquez@pucp.pe

**SUPPLEMENTARY MATERIAL**

**Index**

**Section A1**

**GHG emissions – Characterization factors**

………p.3

**Section A2**

**Pedigree Matrix – Converting qualitative information into quantitative data**

……p.8

**References**

……p.13

**Section A1**

**GHG emissions – Characterization factors**

Table A1 shows the characterization factors used for greenhouse gases in the IPCC 2013 Global Warming Potential (GWP) method (IPCC, 2013).

**Table A1.** Characterization factors for greenhouse gases (GHGs) included in the IPCC 2013 GWP method.

| **Substance** | **CAS** | **Characterization factor** |
| --- | --- | --- |
| (E)-1-Chloro-3,3,3-trifluoroprop-1-ene | 102687-65-0 | 1.49 |
| (E)-1,2,3,3,3-Pentafluoroprop-1-ene | 0 5595-10-8 | 0.079 |
| (Perfluorobutyl)ethylene | 019430-93-4 | 0.136 |
| (Perfluoroctyl)ethylene | 021652-58-4 | 0.0929 |
| (Perfluorohexyl)ethylene | 025291-17-2 | 0.108 |
| (Z)-1,1,1,4,4,4-Hexafluorobut-2-ene | 000692-49-9 | 1.68 |
| (Z)-1,2,3,3,3-Pentafluoroprop-1-ene | 005528-43-8 | 0.233 |
| (Z)-1,3,3,3-Tetrafluoroprop-1-ene | 029118-25-0 | 0.285 |
| 1-Undecanol, 3,3,4,4,5,5,6,6,7,7,8,8,9,9,10,10,11,11,11-nonadecafluoro- | 087017-97-8 | 0.69 |
| 1,1,1,3,3,3-Hexafluoropropan-2-ol | 000920-66-1 | 182 |
| 1,2,2-Trichloro-1,1-difluoroethane | 000354-21-2 | 59.21 |
| 2,3,3,3-Tetrafluoropropene | 000754-12-1 | 0.352 |
| Acetate, 1,1-difluoroethyl 2,2,2-trifluoro- |  | 30.84 |
| Acetate, 2,2,2-trifluoroethyl 2,2,2-trifluoro- | 000407-38-5 | 6.84 |
| Acetate, difluoromethyl 2,2,2-trifluoro- | 002024-86-4 | 27.06 |
| Acetate, methyl 2,2-difluoro- | 000433-53-4 | 3.27 |
| Acetate, methyl 2,2,2-trifluoro- | 000431-47-0 | 52.35 |
| Acetate, perfluorobutyl- | 209597-28-4 | 1.66 |
| Acetate, perfluoroethyl- | 343269-97-6 | 2.06 |
| Acetate, perfluoropropyl- |  | 1.73 |
| Acetate, trifluoromethyl- | 074123-20-9 | 2.07 |
| Butane, 1,1,1,2,2,3,3,4,4-nonafluoro-, HFC-329p | 00375-17-7 | 2355 |
| Butane, 1,1,1,2,2,3,3,4,4-nonafluoro-4-methoxy- | 163702-07-6 | 421 |
| Butane, 1,1,1,3,3-pentafluoro-, HFC-365mfc | 000406-58-6 | 804 |
| Butane, perfluoro- | 000355-25-9 | 9202 |
| Butane, perfluorocyclo-, PFC-318 | 000115-25-3 | 9545 |
| Butanol, 2,2,3,3,4,4,4-heptafluoro- | 000375-01-9 | 33.75 |
| Butanol, 2,2,3,3,4,4,4-heptafluoro-1- | 000375-01-9 | 16.29 |
| Butanol, 2,2,3,4,4,4-hexafluoro-1- | 000382-31-0 | 17.01 |
| Carbon dioxide | 000124-38-9 | 1 |
| Carbon dioxide, fossil | 000124-38-9 | 1 |
| Carbon dioxide, land transformation | 000124-38-9 | 1 |
| Chloroform | 000067-66-3 | 16.4 |
| Cis-perfluorodecalin | 060433-11-6 | 7236 |
| Decane, 1,1,3,3,4,4,6,6,7,7,9,9,10,10,12,12-hexadecafluoro-2,5,8,11-tetraoxado- | 173350-37-3 | 2853 |

**Table A1.** Characterization factors for greenhouse gases (GHGs) included in the IPCC 2013 GWP method (cont.).

| Decane, 1,1,3,3,5,5,7,7,8,8,10,10-dodecafluoro-2,4,6,9-tetraoxa- | 249932-26-1 | 3888 |
| --- | --- | --- |
| Decane, 1,1,3,3,5,5,7,7,9,9-decafluoro-2,4,6,8-tetraoxanonane- | 188690-77-9 | 7333 |
| Decane, 3,3,4,4,6,6,7,7,9,9,10,10-dodecafluoro-2,5,8,11-tetraoxado- | 485399-48-2 | 221 |
| Dinitrogen monoxide | 010024-97-2 | 265 |
| EPTE-furan | 920979-28-8 | 55.54 |
| Ethane, 1-(difluoromethoxy)-1,1,2,2-tetrafluoro- | 032778-11-3 | 4242 |
| Ethane, 1-chloro-1,1-difluoro-, HCFC-142b | 000075-68-3 | 1982 |
| Ethane, 1-chloro-2,2,2-trifluoro-(difluoromethoxy)-, HCFE-235da2 | 026675-46-7 | 491 |
| Ethane, 1-ethoxy-1,1,2,2,2-pentafluoro- | 022052-81-9 | 58.38 |
| Ethane, 1,1'-oxybis[2-(difluoromethoxy)-1,1,2,2-tetrafluoro- | 205367-61-9 | 4916 |
| Ethane, 1,1-dichloro-1-fluoro-, HCFC-141b | 001717-00-6 | 782 |
| Ethane, 1,1-dichloro-1,2-difluoro-, HCFC-132c | 001842-05-3 | 338 |
| Ethane, 1,1-difluoro-, HFC-152a | 000075-37-6 | 138 |
| Ethane, 1,1,1-trichloro-, HCFC-140 | 000071-55-6 | 160 |
| Ethane, 1,1,1-trifluoro-, HFC-143a | 000420-46-2 | 4804 |
| Ethane, 1,1,1-trifluoro-2-bromo- | 000421-06-7 | 173 |
| Ethane, 1,1,1,2-tetrafluoro-, HFC-134a | 000811-97-2 | 1301 |
| Ethane, 1,1,1,2-tetrafluoro-2-bromo-, Halon 2401 | 000124-72-1 | 184 |
| Ethane, 1,1,2-trichloro-1,2-difluoro-, HCFC-122a | 000354-15-4 | 258 |
| Ethane, 1,1,2-trichloro-1,2,2-trifluoro-, CFC-113 | 000076-13-1 | 5824 |
| Ethane, 1,1,2-trifluoro-, HFC-143 | 000430-66-0 | 328 |
| Ethane, 1,1,2,2-tetrafluoro-, HFC-134 | 000359-35-3 | 1116 |
| Ethane, 1,1,2,2-tetrafluoro-1-(fluoromethoxy)- | 037031-31-5 | 871 |
| Ethane, 1,1,2,2-tetrafluoro-1-methoxy-2-(1,1,2,2-tetrafluoro-2-methoxyethoxy)- | 485399-46-0 | 236 |
| Ethane, 1,1,2,2-tetrafluoro-1,2-dimethoxy- | 073287-23-7 | 222 |
| Ethane, 1,2-dibromotetrafluoro-, Halon 2402 | 000124-73-2 | 1472 |
| Ethane, 1,2-dichloro- | 000107-06-2 | 0.898 |
| Ethane, 1,2-dichloro-1,1,2-trifluoro-, HCFC-123 | 000354-23-4 | 370 |
| Ethane, 1,2-dichloro-1,1,2,2-tetrafluoro-, CFC-114 | 000076-14-2 | 8592 |
| Ethane, 1,2-difluoro-, HFC-152 | 000624-72-6 | 16.41 |
| Ethane, 2-chloro-1,1,1,2-tetrafluoro-, HCFC-124 | 002837-89-0 | 527 |
| Ethane, 2-chloro-1,1,2-trifluoro-1-methoxy- | 000425-87-6 | 122 |
| Ethane, 2,2-dichloro-1,1,1-trifluoro-, HCFC-123 | 000306-83-2 | 79.37 |
| Ethane, chloropentafluoro-, CFC-115 | 000076-15-3 | 7665 |
| Ethane, fluoro-, HFC-161 | 000353-36-6 | 3.64 |
| Ethane, hexafluoro-, HFC-116 | 000076-16-4 | 11123 |
| Ethane, pentafluoro-, HFC-125 | 000354-33-6 | 3169 |
| Ethanol, 2-fluoro- | 000371-62-0 | 0.88 |
| Ethanol, 2,2-difluoro- | 000359-13-7 | 3.03 |
| Ethanol, 2,2,2-trifluoro- | 000075-89-8 | 19.95 |
| Ethene, 1,1-difluoro-, HFC-1132a | 000075-38-7 | 0.0422 |
| Ethene, 1,1,2-trifluoro-2-(trifluoromethoxy)- | 001187-93-5 | 0.209 |

**Table A1.** Characterization factors for greenhouse gases (GHGs) included in the IPCC 2013 GWP method (cont.).

| Ether, 1,1,1-trifluoromethyl methyl-, HFE-143a | 000421-14-7 | 523 |
| --- | --- | --- |
| Ether, 1,1,2,2-Tetrafluoroethyl 2,2,2-trifluoroethyl-, HFE-347mcf2 | 000406-78-0 | 854 |
| Ether, 1,1,2,2-Tetrafluoroethyl 2,2,2-trifluoroethyl-, HFE-347pcf2 | 000406-78-0 | 889 |
| Ether, 1,1,2,2-Tetrafluoroethyl methyl-, HFE-254cb2 | 000425-88-7 | 301 |
| Ether, 1,1,2,3,3,3-Hexafluoropropyl methyl-, HFE-356mec3 | 000382-34-3 | 387 |
| Ether, 1,1,2,3,3,3-Hexafluoropropyl methyl-, HFE-356pcc3 | 000382-34-3 | 413 |
| Ether, 1,1,2,3,3,3-Hexafluoropropyl methyl-, HFE-356pcf2 | 000382-34-3 | 719 |
| Ether, 1,1,2,3,3,3-Hexafluoropropyl methyl-, HFE-356pcf3 | 000382-34-3 | 446 |
| Ether, 1,2,2-trifluoroethyl trifluoromethyl-, HFE-236ea2 | 084011-06-3 | 1243 |
| Ether, 1,2,2-trifluoroethyl trifluoromethyl-, HFE-236fa | 084011-06-3 | 979 |
| Ether, 2-chloro-1,1,2-trifluoroethyl difluoromethyl-, HCFE-235ca2 (enflurane) | 013838-16-9 | 583 |
| Ether, 2,2,3,3,3-Pentafluoropropyl methyl-, HFE-365mcf3 | 000378-16-5 | 0.928 |
| Ether, bis(2,2,2-trifluoroethyl)- | 000333-36-8 | 16.76 |
| Ether, di(difluoromethyl), HFE-134 | 001691-17-4 | 5564 |
| Ether, difluoromethyl 1,2,2,2-tetrafluoroethyl-, HFE-236ea2 (desflurane) | 057041-67-5 | 1792 |
| Ether, difluoromethyl 2,2,2-trifluoroethyl-, HFE-245cb2 | 001885-48-9 | 654 |
| Ether, difluoromethyl 2,2,2-trifluoroethyl-, HFE-245fa1 | 001885-48-9 | 828 |
| Ether, difluoromethyl 2,2,2-trifluoroethyl-, HFE-245fa2 | 001885-48-9 | 812 |
| Ether, ethyl 1,1,2,2-tetrafluoroethyl-, HFE-374pc2 | 000512-51-6 | 627 |
| Ether, ethyl trifluoromethyl-, HFE-263m1 | 000690-22-2 | 29.46 |
| Ether, nonafluorobutane ethyl-, HFE569sf2 (HFE-7200) | 163702-05-4 | 56.82 |
| Ether, pentafluoromethyl-, HFE-125 | 003822-68-2 | 12403 |
| Fluoridate, 1,1-difluoroethyl carbono- |  | 26.82 |
| Fluoridate, methyl carbono- | 001538-06-3 | 95.33 |
| Fluoroxene | 000406-90-6 | 0.0542 |
| Formate, 1,1,1,3,3,3-hexafluoropropan-2-yl- | 856766-70-6 | 333 |
| Formate, 1,2,2,2-tetrafluoroethyl- | 481631-19-0 | 470 |
| Formate, 2,2,2-trifluoroethyl- | 032042-38-9 | 33.44 |
| Formate, 3,3,3-trifluoropropyl- |  | 17.4 |
| Formate, perfluorobutyl- | 197218-56-7 | 392 |
| Formate, perfluoroethyl- | 313064-40-3 | 580 |
| Formate, perfluoropropyl- | 271257-42-2 | 376 |
| Formate, trifluoromethyl- | 085358-65-2 | 588 |
| Halothane | 000151-67-7 | 41.11 |
| Heptanol, 3,3,4,4,5,5,6,6,7,7,7-undecafluoro- | 185689-57-0 | 0.426 |
| Hexane, perfluoro- | 000355-42-0 | 7912 |
| HFE-227EA | 002356-62-9 | 6452 |
| HFE-236ca12 (HG-10) | 078522-47-1 | 5353 |
| HFE-263fb2 | 000460-43-5 | 1.32 |
| HFE-329mcc2 | 067490-36-2 | 3067 |
| HFE-338mcf2 | 156053-88-2 | 929 |
| HFE-338pcc13 (HG-01) | 188690-78-0 | 2908 |

**Table A1.** Characterization factors for greenhouse gases (GHGs) included in the IPCC 2013 GWP method (cont.).

| HFE-43-10pccc124 (H-Galden1040x) |  | 2817 |
| --- | --- | --- |
| Methane | 000074-82-8 | 28 |
| Methane, (difluoromethoxy)((difluoromethoxy)difluoromethoxy)difluoro- | 249932-25-0 | 5303 |
| Methane, biogenic | 000074-82-8 | 25.25 |
| Methane, bromo-, Halon 1001 | 000074-83-9 | 2.35 |
| Methane, bromochlorodifluoro-, Halon 1211 | 000353-59-3 | 1746 |
| Methane, bromodifluoro-, Halon 1201 | 001511-62-2 | 376 |
| Methane, bromotrifluoro-, Halon 1301 | 000075-63-8 | 6292 |
| Methane, chlorodifluoro-, HCFC-22 | 000075-45-6 | 1765 |
| Methane, chlorotrifluoro-, CFC-13 | 000075-72-9 | 13893 |
| Methane, dibromo- | 000074-95-3 | 1.01 |
| Methane, dibromodifluoro-, Halon 1202 | 000075-61-6 | 231 |
| Methane, dichloro-, HCC-30 | 000075-09-2 | 8.92 |
| Methane, dichlorodifluoro-, CFC-12 | 000075-71-8 | 10239 |
| Methane, dichlorofluoro-, HCFC-21 | 000075-43-4 | 148 |
| Methane, difluoro-, HFC-32 | 000075-10-5 | 677 |
| Methane, difluoro(fluoromethoxy)- | 000461-63-2 | 617 |
| Methane, difluoro(methoxy)- | 000359-15-9 | 144 |
| Methane, fluoro-, HFC-41 | 000593-53-3 | 116 |
| Methane, fluoro(fluoromethoxy)- | 000462-51-1 | 130 |
| Methane, fluoro(methoxy)- | 000460-22-0 | 12.56 |
| Methane, fossil | 000074-82-8 | 28 |
| Methane, monochloro-, R-40 | 000074-87-3 | 12.18 |
| Methane, tetrachloro-, CFC-10 | 000056-23-5 | 1728 |
| Methane, tetrafluoro-, CFC-14 | 000075-73-0 | 6626 |
| Methane, trichlorofluoro-, CFC-11 | 000075-69-4 | 4663 |
| Methane, trifluoro-, HFC-23 | 000075-46-7 | 12398 |
| Methane, trifluoro(fluoromethoxy)- | 002261-01-0 | 751 |
| Methyl perfluoroisopropyl ether | 022052-84-2 | 363 |
| Nitrogen fluoride | 007783-54-2 | 16070 |
| Nonanol, 3,3,4,4,5,5,6,6,7,7,8,8,9,9,9-pentadecafluoro- | 000755-02-2 | 0.327 |
| Octa deca fluoro octane | 000307-34-6 | 7620 |
| Pentafluorobutene-1 | 000374-27-6 | 0.126 |
| Pentane, 2,3-dihydroperfluoro-, HFC-4310mee | 138495-42-8 | 1647 |
| Pentane, perfluoro- | 000678-26-2 | 8547 |
| Pentanol, 2,2,3,3,4,4,5,5-octafluorocyclo- | 016621-87-7 | 12.85 |
| Pentanone, 1,1,1,2,2,4,5,5,5-nonafluoro-4-(trifluoromethyl)-3- | 000756-13-8 | 0.0997 |
| Perfluorobut-1-ene | 000357-26-6 | 0.0914 |
| Perfluorobut-2-ene | 000360-89-4 | 1.76 |
| Perfluorobuta-1,3-diene | 000685-63-2 | 0.00359 |
| Perfluorocyclopentene | 000559-40-0 | 1.86 |
| Perfluorodecalin (mixed) | 000306-94-5 | 7185 |
| Perfluorodecalin (trans) | 060433-12-7 | 6288 |
| Perfluoroheptane | 000335-57-9 | 7822 |
| Perfluoropropene | 000116-15-4 | 0.07 |

**Table A1.** Characterization factors for greenhouse gases (GHGs) included in the IPCC 2013 GWP method (cont.).

| PFPMIE |  | 9706 |
| --- | --- | --- |
| Propanal, 3,3,3-trifluoro- | 000460-40-2 | 0.0108 |
| Propane, 1-ethoxy-1,1,2,2,3,3,3-heptafluoro | 022052-86-4 | 60.63 |
| Propane, 1-ethoxy-1,1,2,3,3,3-hexafluoro- | 000380-34-7 | 23.35 |
| Propane, 1,1,1-trifluoro-, HFC-263fb | 000421-07-8 | 75.52 |
| Propane, 1,1,1,2,2-pentafluoro-, HFC-245cb | 001814-88-6 | 4622 |
| Propane, 1,1,1,2,2,3-hexafluoro-, HFC-236cb | 000677-56-5 | 1207 |
| Propane, 1,1,1,2,2,3,3-heptafluoro-, HFC-227ca | 002252-84-8 | 2642 |
| Propane, 1,1,1,2,2,3,3-heptafluoro-3-(1,2,2,2-tetrafluoroethoxy)- | 003330-15-2 | 6487 |
| Propane, 1,1,1,2,3-pentafluoro-, HFC-245eb | 000431-31-2 | 290 |
| Propane, 1,1,1,2,3,3-hexafluoro-, HFC-236ea | 000431-63-0 | 1335 |
| Propane, 1,1,1,2,3,3-hexafluoro-3-(trifluoromethoxy)-, HFE-329me3 | 428454-68-6 | 4550 |
| Propane, 1,1,1,2,3,3,3-heptafluoro-, HFC-227ea | 000431-89-0 | 3348 |
| Propane, 1,1,1,3,3-pentafluoro-, HFC-245fa | 000460-73-1 | 858 |
| Propane, 1,1,1,3,3,3-hexafluoro-, HCFC-236fa | 000690-39-1 | 8056 |
| Propane, 1,1,1,3,3,3-Hexafluoro-2-(difluoromethoxy) | 026103-08-2 | 2621 |
| Propane, 1,1,1,3,3,3-hexafluoro-2-(fluoromethoxy)- | 028523-86-6 | 216 |
| Propane, 1,1,1,3,3,3-hexafluoro-2-methoxy-(9CI) | 013171-18-1 | 13.58 |
| Propane, 1,1,2,2-tetrafluoro-3-methoxy- | 060598-17-6 | 0.525 |
| Propane, 1,1,2,2,3-pentafluoro-, HFC-245ca | 000679-86-7 | 716 |
| Propane, 1,1,2,3,3-pentafluoro-, HFC-245ea | 024270-66-4 | 235 |
| Propane, 1,3-dichloro-1,1,2,2,3-pentafluoro-, HCFC-225cb | 000507-55-1 | 525 |
| Propane, 2-(difluoromethoxymethyl)-1,1,1,2,3,3,3-heptafluoro- | 163702-08-7 | 116 |
| Propane, 2,2-difluoro-, HFC-272ca | 000420-45-1 | 144 |
| Propane, 3,3-dichloro-1,1,1,2,2-pentafluoro-, HCFC-225ca | 000422-56-0 | 127 |
| Propane, perfluoro- | 000076-19-7 | 8900 |
| Propane, perfluorocyclo- |  | 9198 |
| Propane,1,1,1,2,2,3,3-heptafluoro-3-methoxy-, HFE-347mcc3 (HFE-7000) | 000375-03-1 | 530 |
| Propanol, 2,2,3,3-tetrafluoro-1- | 000076-37-9 | 12.99 |
| Propanol, 3,3,3-trifluoro-1- | 002240-88-2 | 0.39 |
| Propanol, pentafluoro-1- | 000422-05-9 | 18.8 |
| Sulfur hexafluoride | 002551-62-4 | 23507 |
| Sulfuryl fluoride | 002699-79-8 | 4095 |
| Tetrafluoroethylene | 000116-14-3 | 0.00292 |
| trans-1,3,3,3-Tetrafluoropropene | 001645-83-6 | 0.953 |
| Trifluorobutanol | 000461-18-7 | 0.0189 |
| Trifluoroethyl acetate | 000383-63-1 | 1.37 |
| Trifluoromethylsulfur pentafluoride | 000373-80-8 | 17449 |
| Trifluoropropene, HFC-1243zf | 000677-21-4 | 0.149 |
| Vinylfluoride | 000075-02-5 | 0.0168 |

**Section A2**

**Pedigree Matrix – Converting qualitative information into quantitative data**

The Pedigree matrix developed by Weidema and Wesnaes (1996) and Weidema (1998) allows converting qualitative information used in life cycle modelling into quantitative information. The use of this matrix is based on the fact that the GHG emissions per food product can be described using lognormal distributions, in which the mean GHG emission value used is considered the geometric mean of the distribution, whereas the geometric standard deviation is calculated as an outcome of computing the Pedigree matrix. In this sense, data sources are assessed according to six items:

- Reliability.
- Completeness.
- Temporal correlation.
- Geographical correlation.
- Further technological correlation.
- Sample size

Each of these items is then divided into five quality levels, as described in Table A2. The mathematical computation of each level in terms of calculating the geometric variance is shown in Table A3. An additional item is included in the equation shown in the main manuscript to account for the uncertainty factor (U_b_) linked to the environmental burden (see Table A4), which in this case will always be climate change.

Once the formula shown is computed, as presented below, the variance is transformed into standard deviation. Thereafter, two uncertainty levels (high and low) are calculated based on the geometric standard deviation factor that was computed.

$\sigma_{g}^{2}=\exp\sqrt{\left( \ln U_{1} \right)^{2}+\left( \ln U_{2} \right)^{2}+\left( \ln U_{3} \right)^{2}+\left( \ln U_{4} \right)^{2}+{\left( \ln U_{5} \right)^{2}+\left( \ln U_{6} \right)^{2}+\left( \ln U_{b} \right)}^{2}}$ [eq. 1]

**Table A2.** Pedigree matrix used to evaluate the quality of data sources. Adapted from Weidema and Wesnaes (1996) and Weidema (1998).

| **Indicator score** | **1** | **2** | **3** | **4** | **5 (default)** |
| --- | --- | --- | --- | --- | --- |
| **Reliability** | Verified data base on measurements | Verified data partly based on assumptions or non-verified data based on measurements | Non-verified data partly based on qualified estimates | Qualified estimate (e.g., by industrial expert) | Non-qualified estimate |
| **Completeness** | Representative data from all sites relevant for the market considered, over an adequate period to even out normal fluctuations | Representative data from >50% of the sites relevant for the market considered, over an adequate period to even out normal fluctuations | Representative data from only some sites (<<50%) relevant for the market considered *or* >50% of sites but from shorter periods | Representative data from only one site relevant for the market considered *or* some sites but from shorter periods | Representativeness unknown or data from a small number of sites *and* from shorter periods |
| **Temporal correlation** | Less than 3 years of difference to the time period of the dataset | Less than 6 years of difference to the time period of the dataset | Less than 10 years of difference to the time period of the dataset | Less than 15 years of difference to the time period of the dataset | Age of data unknown or more than 15 years of difference to the time period of the dataset |
| **Geographical correlation** | Data from area under study | Average data from larger area in which the area under study is included | Data from area with similar production conditions | Data from area with slightly similar production conditions | Data from unknown or distinctly different area (South America instead of Middle East; Ukraine instead of Baltic states) |
| **Further technological correlation** | Data from enterprises, processes and materials under study | Data from processes and materials under study (i.e., identical technology) but from different enterprises | Data from processes and materials under study but from different technology | Data on related processes and materials | Data on related processes on laboratory scale or from different technology |
| **Sample size** | >100, continuous measurement, balance of purchased products | >20 | >10, aggregated in environmental report | >=3 | unknown |

**Table A3.** Uncertainty factors for the Pedigree Matrix scores.

| **Indicator score** | **1** | **2** | **3** | **4** | **5** |
| --- | --- | --- | --- | --- | --- |
| **Reliability** | 1 | 1.05 | 1.10 | 1.20 | 1.50 |
| **Completeness** | 1 | 1.02 | 1.05 | 1.10 | 1.20 |
| **Temporal correlation** | 1 | 1.03 | 1.10 | 1.20 | 1.50 |
| **Geographical correlation** | 1 | 1.01 | 1.02 | -- | 1.10 |
| **Further technological correlation** | 1 | -- | 1.20 | 1.50 | 2.00 |
| **Sample size** | 1 | 1.02 | 1.05 | 1.10 | 1.20 |

**Table A4.** Uncertainty factors considered for environmental impacts in the Pedigree matrix (U_b_).

| **Impact** | **Unit** | **Uncertainty factor (U_b_)** |
| --- | --- | --- |
| **Acidification potential** | kg SO_2_eq | 1.05 |
| **Global Warming Potential** | kg CO_2_eq | **1.05** |
| **Eutrophication potential** | kg N eq | 1.50 |
| **Freshwater aquatic eco-toxicity** | kg 1.4-DCBeq | 2.00 |
| **Land use** | m^2^a | 1.50 |
| **Marine aquatic eco-toxicity** | kg 1.4-DCBeq | 2.00 |
| **Photochemical oxidation** | kg formed ozone | 1.50 |
| **Stratospheric ozone depletion** | kg CFC-11eq | 1.50 |
| **Terrestrial eco-toxicity** | kg 1.4-DCBeq | 2.00 |

*Case study for bananas*

The GHG emissions value used for banana was retrieved from the Life Cycle Inventories reported in Roibás et al. (2016), a peer reviewed article that analyzed, using a life-cycle perspective, the GHG emissions of the production of bananas in Ecuador. The reliability of the data reported was quantified in level 2 (U_1_= 1.05). Although the authors report that the data used was verified based on measurements, they also report an important number of assumptions. Hence, a precautionary factor from level 2 was selected rather than level 1. In terms of completeness (item 2), a factor of 1.20 was used as a precautionary value (level 5), since little is known about the representativeness of the sites measured in Roibás et al. (2016) regarding the market considered. In terms of temporal correlation (U_3_), the data used for this study were from year 2010. Hence, this represents less than a 6 year timespan to the period assessed. Based on the description provided in Tables A2 and A3, this item was quantified in level 2 (U_3_= 1.02). The fourth item is geographical correlation. Ecuador is considered to have similar cultivation conditions in Amazon areas for the production of banana as Peru. Hence, this item was quantified in level 3 (U_4_= 1.02). Item number 5 is defined as further technological correlation. Considering that the technology to produce bananas in the Amazon basin is relatively uniform a level 2 factor was chosen (U_5_= 1.20). Finally, the sixth item assessed takes into account the sample size (U_6_) that is used to compute the GHG emissions. In this particular case, the sample size added up to 17 sites that were inventoried. Therefore, a factor of 1.02, linked to level 2 of uncertainty in this item, was fixed. A seventh item is related to the inherent uncertainty of the environmental impact assessed, in this case climate change (U_b_= 1.05), based on Weidema and Wesnaes (1996) and Weidema (1998).

**References**

IPCC. Climate Change 2013. The Physical Science Basis. Working Group I contribution to the 5th Assessment Report of the IPCC. November 2013. Intergovernamental Panel on Climate Change. Available from: <http://www.climatechange2013.org>.

Roibás L, Elbehri A, Hospido A. Carbon footprint along the Ecuadorian banana supply chain: Methodological improvements and calculation tool. J. Clean. Prod. 2016;112: 2441-2451.

Weidema BP, Wesnaes MS. Data quality management for life cycle inventories—an example of using data quality indicators. J Clean Prod. 1996;4(3-4): 167-174.

Weidema BP. Multi-user test of the data quality matrix for product life cycle inventory data. Int J Life Cycle Assess. 1998;3(5): 259-265.
